# Solid surface frustrated Lewis pair constructed on layered AlOOH for hydrogenation reaction

**DOI:** 10.1038/s41467-022-29970-6

**Published:** 2022-04-28

**Authors:** Shulin Liu, Minghua Dong, Yuxuan Wu, Sen Luan, Yu Xin, Juan Du, Shaopeng Li, Huizhen Liu, Buxing Han

**Affiliations:** 1 Beijing National Laboratory for Molecular Sciences, CAS Key Laboratory of Colloid and Interface and Thermodynamics, Institute of Chemistry, Chinese Academy of Sciences, Beijing, 100190 China; 2 School of Chemistry and Chemical Engineering, University of Chinese Academy of Sciences, Beijing, 100049 China

**Keywords:** Heterogeneous catalysis, Catalytic mechanisms, Chemical engineering

## Abstract

Designing heterogeneous solid surface frustrated Lewis pair (ssFLP) catalyst for hydrogenation is a new challenge in catalysis and no research has been reported on the construction of ssFLP on boehmite (AlOOH) surfaces up to now as far as we know. Herein, AlOOH with a layer structure is prepared and it is found that the Lewis basic O_Hv_ site (one H removed from OH) and an adjacent Lewis acidic unsaturated Al site (Al^3+^_unsatur_.) proximal to a surface OH_v_ (OH vacancy) on AlOOH layers could form the ssFLP. The layered structure of AlOOH and its abundant OH defects over the surface result in a high concentration of O_Hv_/Al^3+^_unsatur_. FLPs, which are conducive to highly efficient hydrogen activation for hydrogenation of olefins and alkynes with low H-H bond dissociates activation energy of 0.16 eV under mild conditions (T = 80°C and P(H_2_) = 2.0 MPa). This work develops a new kind of hydrogenation catalyst and provides a new perspective for creating solid surface FLP.

## Abstract (web summary)

Designing heterogeneous solid surface frustrated Lewis pair (ssFLP) catalyst for hydrogenation is a new challenge in catalysis. Here, the authors show the ssFLP can be constructed on layered AlOOH for hydrogenation reactions under mild conditions.

## Introduction

Hydrogen activation is the key step in catalytic hydrogenation reaction, which is very important in the petrochemical industry. The process has been well developed over transition metal complexes, metal-based heterogeneous catalysts^1^ and nonmetallic N, S, B, or P heteroatom doped carbon-based catalyst^2–6^. In 2006, W. Stephan group^7^ firstly reported a novel mode for H_2_ activation that the frustrated Lewis pair (FLP) could activate H_2_ and an appropriate distance between Lewis acid and base sites is critical^8^. The early research of FLP was based on molecular-based homogeneous complex^9–12^, such as phosphide–boranes, which suffers the difficulties in catalyst recovery and product purification. Therefore, it is more expected to develop heterogeneous catalysts with FLP-like activity^13,14^. The construction of surface FLP over noble-metal-based heterogeneous catalysts has been successfully demonstrated. For example, the formation of FLP consisting of sodium hydride (Na^+^H^−^) and a framework-bound hydroxy proton O(H^+^) is reported upon H_2_ treatment of zeolite NaY loaded with Pt nanoparticles (Pt_x_/NaY)^15^. The Au powders and molecular Lewis bases (for example, imine and nitrile) also could form FLP^16^.

It is well known that it is easy to construct defects on reducible oxides, beneficial from oxygen vacancies. Recently, solid surface FLP sites on reducible metal oxide CeO_2_ were developed by Qu et. al.^17^ since its reversible Ce^3+^/Ce^4+^ redox pair and rich surface defects chemistry. It was found that the richness of surface defects is the key to the construction of surface Lewis acidic center by two adjacent reduced surface Ce atoms near an oxygen vacancy and the neighboring surface lattice oxygen was Lewis base. Ozin and co-workers^18–21^ developed the surface FLP sites over In_2_O_3-x_(OH)_y_ composed of a Lewis basic surface OH adjacent to a Lewis acid indium proximal to an oxygen vacancy, which assisted the adsorption and heterolytic dissociation of H_2_ in the reverse water gas shift (RWGS) reaction in photocatalysis. Ni-O Lewis pair at the NiO_x_-Ni interface was also fabricated for the activation of water^22^. Obviously, the rational design and controllable construction of specific Lewis pairs with high catalytic performance are now limited to a few specific oxide systems through the formation of surface oxygen vacancies or construction of hydroxyl-metal ion pairs. The development of novel ssFLPs is very interesting from both scientific and practical perspectives and yet it is still a great challenge to construct an efficient ssFLPs site on inert metal oxide for hydrogenation reaction.

Boehmite (AlOOH), as one of the main components of bauxite ore, is abundant in natural resources^23^ and has been studied extensively for various applications^24–28^, such as adsorbents, porous materials, catalyst support. Especially, AlOOH is a main-group metal hydroxide and non-reducible. Herein, we prepared a series of layered AlOOH, where a novel ssFLPs site was found. With these layered AlOOH, the hydrogenation of olefins and alkynes could proceed efficiently under mild conditions (T = 80°C and P(H_2_) = 2.0 MPa). It was found that the abundant OH vacancies (OH_v_) are the key to the construction of the Lewis acid center. The ssFLPs were created by a Lewis basic O_Hv_ site and an adjacent Lewis acidic unsaturated Al site (Al^3+^_unsatur._) proximal to a surface OH vacancy on AlOOH layers. The unique layer structure of AlOOH and abundant OH defects result in a high concentration of O_Hv_ /Al^3+^_unsatur._ FLPs, which are conducive to highly efficient hydrogen activation.

## Results and discussion

### Structural characterizations

**Fig. 1:**
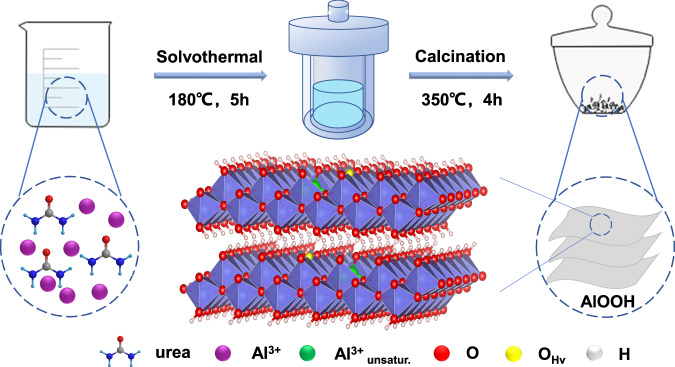
Catalyst preparation. Schematic process for the fabrication of AlOOH catalyst.

The catalysts were prepared using Al(NO_3_)_3_·9H_2_O as the precursor and urea as the precipitant by a solvothermal method at 180°C for 5 h, as shown in Fig. 1. When the mole ratio of urea and Al(NO_3_)_3_·9H_2_O was 1:1, the catalyst was denoted as AlOx-U1. When the mole ratio of urea and Al(NO_3_)_3_·9H_2_O was above 1:1, the catalyst was denoted as AlOOH-Ux and x stands for the mole ratio of Al and urea used.

**Fig. 2:**
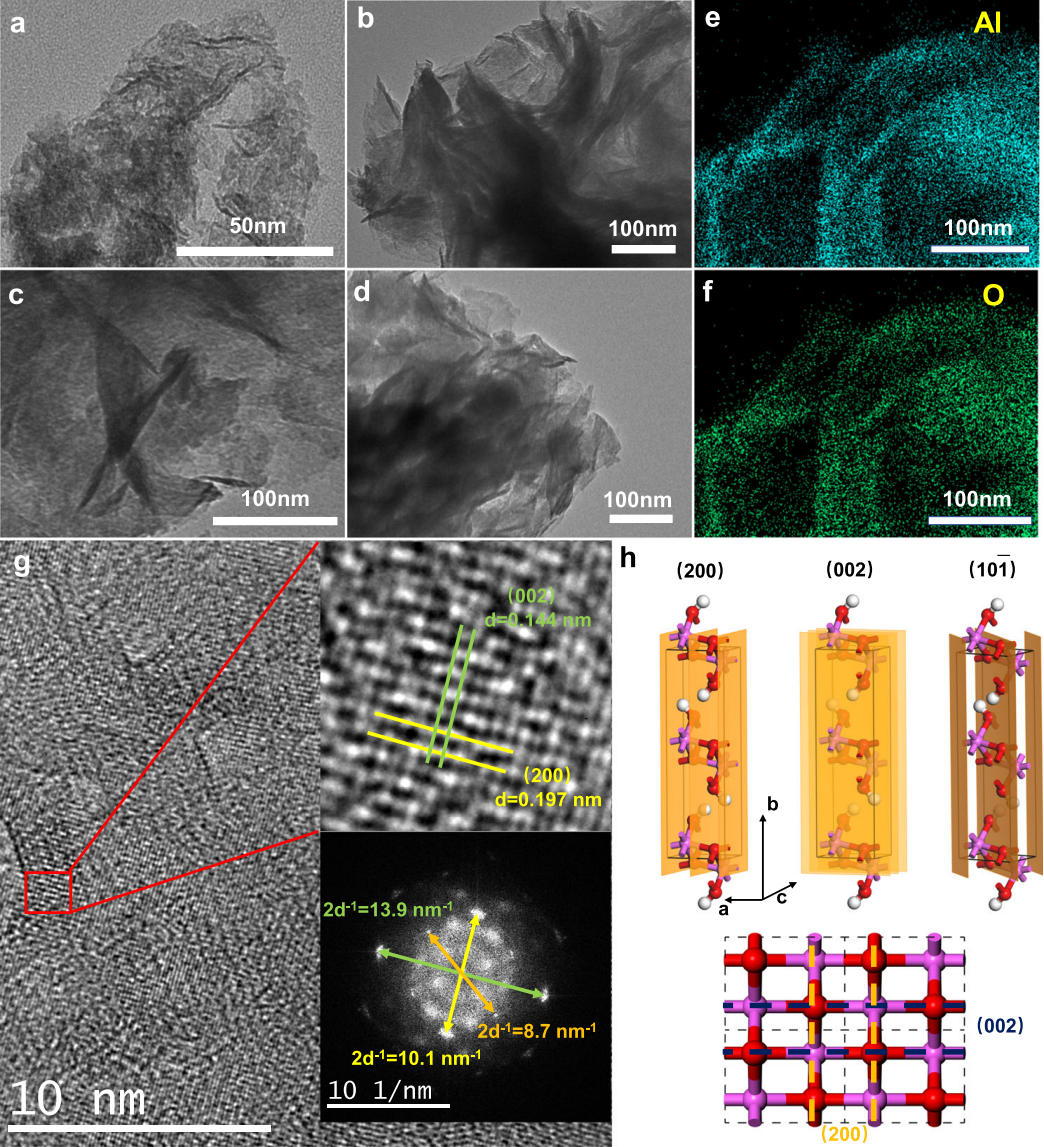
TEM and HR-TEM images for catalysts. **a** AlOx-U1. **b** AlOOH-U2. **c** AlOOH-U3. **d** AlOOH-U4. **e** Al and **f** O EDS elemental mapping images of the AlOOH-U2. **g** HR-TEM images of the AlOOH-U2 and the illustration as FFT images. **h** Crystal plane models of (200), (002), (10-1).

**Fig. 3:**
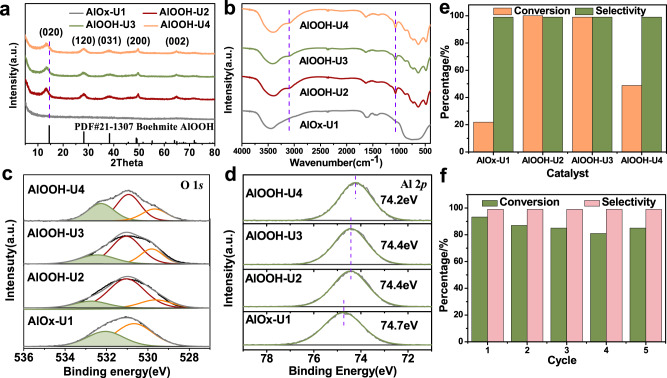
Characterization and activity of the AlOOH catalyst. **a** XRD **b** FTIR **c** O1s and **d** Al 2p XPS and **e** Reactivity comparison of different catalyst. **f** Catalyst recycling of AlOOH-U2. Catalyst (50 mg), temperature (80 °C), H_2_ pressure (2 MPa), time (6 h), styrene (0.5 mmol), THF (2 mL), stirring speed (600 rpm) and n-decane as internal standard.

Transmission electron microscopy (TEM) images are shown in Fig. 2. AlOOH-U2, AlOOH-U3 and AlOOH-U4 presented layered structure, while AlOx-U1 showed the initial formation state of the layer due to the lower OH^−^ concentration from urea decomposition during the solvothermal reaction. Al and O elements were presented in the EDS mapping images with different colors, meaning successful acquisition of AlOOH. The catalysts were also characterized by powder X-ray diffraction (PXRD) and the results were shown in Fig. 3a. No peaks were observed in the PXRD pattern of AlOx-U1 due to the amorphous nature of AlOx made of a recipe of less urea. AlOOH-U2, AlOOH-U3 and AlOOH-U4 exhibited the typical peaks of orthorhombic AlOOH crystal structure^29^ (JCPDS No. 21-1307), while the weak peak intensities indicated the poor crystallinity^30^. The coexistence of small crystalline areas was shown in the following HR-TEM images (Fig. 2g), and several series of patterns, corresponding to (010) facets, can be found in FFT images. The (200), (002) and (10-1) facets which are orthogonal to the (010) facets can be observed in the images. It demon strates that as-prepared AlOOH exposed (010) facets. Furthermore, the reflection of (020) lattice plane shifted to smaller 2θ angles compared with pristine AlOOH, implying the existence of lattice strain^31,32^. It demonstrates that the preparation method is conducive to the stripping of the catalyst along the [010] direction. The surface energies of different facets were calculated, and (010) facets had the lowest surface energy, as shown in Supplementary Table 1. The expanded lattice spacings of the as-prepared catalysts can be rationalized in terms of the formation of layered structure AlOOH rather than nanosheets. These are also supported by images (Supplementary Fig. 1) of the other two samples AlOOH-U3, AlOOH-U4.

The FTIR spectra of the catalysts are shown in Fig. 3b. A strong and broad band at ca. 3415 cm^−1^ in the spectra of all samples are assigned to the stretching vibration of free OH, probably from hydrogen-bonded hydroxyl groups or adsorbed water^30^. The peak at ca. 1648 cm^−1^ can be attributed to the bending vibration of surface OH or adsorbed water^33^. The peaks at ca. 3099 cm^−1^ and 1075 cm^−1^ in the spectra of AlOOH-Ux are attributed to the stretching and bending vibration modes of -OH_Al-OH_, respectively, further confirming that AlOOH was obtained. However, these two peaks were not observed in the spectrums of AlOx-U1. The band at ca. 738 cm^−1^ is associated with the vibration mode of AlO_6_.

XPS technique was used to analyze the surface state of O elements in the catalysts and the spectra are shown in Fig. 3c. The fitted O1s spectra of AlOOH-U2, AlOOH-U3 and AlOOH-U4 presented three peaks at 529.5–529.8, 530.9–531.1, and 532.3–532.7 eV, attributing to oxygen in lattice structure Al-O-Al (O_L_), hydroxyl bound with Al to form Al-OH (-OH _Al-OH_) or oxygen species (O_ad_, -OH_ad_) adsorbed on the surface OH defects and adsorbed water (O_adH2O_), respectively^33^. As a contrast, the deconvolution of the O1s spectrum for AlOx-U_1_ only gave two peaks at 530.6 eV and 532.1 eV, assigning to the lattice oxygen and surface adsorbed water, which is consistent with the results of FTIR analysis. Compared with AlOx- U1, the Al 2p binding energy (Fig. 3d) for AlOOH-U2, AlOOH-U3 and AlOOH-U4 was lower which was caused by the electron-rich unsaturated Al attributed to OH defects on the surface. In addition, the ratio of O_adH2O_/O_Total_ of AlOx-U1, AlOOH-U2, AlOOH-U3, and AlOOH-U4 are 39.4, 14.5, 21.6, and 32.9% (Supplementary Table 3), respectively, which is related to the catalytic activity, discussed below.

### Catalytic performance

The catalytic performance of the as-prepared catalysts was checked for the hydrogenation of styrene to ethylbenzene, as shown in Fig. 3e. The reaction conditions were optimized as shown in Supplementary Table 2. AlOOH-U2 exhibited the best catalytic performance with a complete conversion and >99% selectivity towards ethylbenzene. Despite the low catalytic activity of AlOOH-U2 observed in the initial 2 h (Supplementary Table 2, entry 5), a satisfying complete hydrogenation of styrene was realized by prolonging the reaction time to 6 h at 80 °C (Supplementary Table 2, entry 7). The reaction process depends upon H_2_ pressure and the conversion increases with the increase of H_2_ pressure (Supplementary Table 2, entry 10–12).

The reusability of AlOOH-U2 was evaluated under the optimized reaction conditions. The catalysts were recovered by centrifugation and washed with THF several times after reaction, and then dried at 60 °C in a vacuum oven overnight for the next use. The AlOOH-U2 can be used at least 5-times without a considerable decrease in activity and selectivity (Fig. 3f). The almost unchanged phase and layered morphology illustrate that the catalyst AlOOH-U2 is stable during hydrogenation reactions (Supplementary Fig. 2b, d). Furthermore, it should be noted that the activity of AlOOH-U2 after calcination in H_2_ flow at 300 °C did not decrease (Table 1, entry 3). The FTIR characteristic peak (ca. 3099 cm^−1^ and 1075 cm^−1^) of AlOOH-U2 still existed after calcination, indicating the catalyst was stable under H_2_ atmosphere even at 300 °C (Supplementary Fig. 3).

**Table 1:** The catalytic activity of AlOOH-U2 with different treatment condition.

Entry	Catalyst	Conv./%	Sel./%
1^***a***^	AlOOH-U2	0	–
2^***b***^	AlOOH-U2	0	–
3^***c***^	AlOOH-U2	0	–
4	AlOOH-U2-H_2_-300 °C	99	>99
5	AlOOH-U2-air-450 °C	17.0	>99
6	AlOOH-U2-Ar-450 °C	100	>99
7^***d***^	AlOOH-U2-Ar-450 °C	100	>99

**Reaction conditions**: Catalyst (50 mg), temperature (80 °C), H_2_ pressure (2 MPa), time (6 h), styrene (0.5 mmol), THF (2 mL), stirring speed (600 rpm) and n-decane as internal standard. *a*: H_2_O (2 mL) as the solvent. *b*: 5 × 10^−3^mol·L^−1^/mg_AlOOH-U2_ pyridine as additive. *c*: 5 × 10^−3^mol·L^−1^/mg_AlOOH-U2_ benzoic acid as additive. *d*: The reaction time was 4 h.

**Fig. 4:**
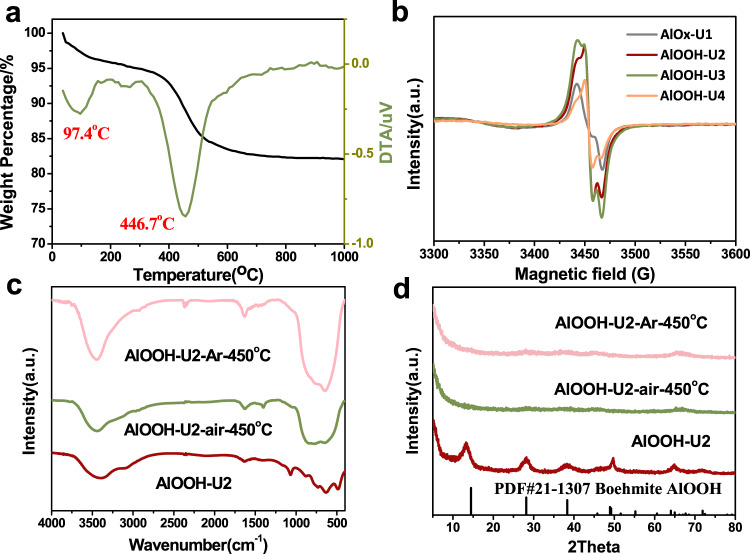
Characterization of the AlOOH catalyst. **a** TGA analysis of AlOOH-U2 catalyst. **b** EPR spectra of different catalysts synthesized by different urea usage. **c** FTIR spectra and **d** XRD patterns of AlOOH-U2 catalyst with different treatment conditions.

Figure 4a shows the thermogravimetric analysis (TGA) curves for AlOOH-U2. There are two obvious steps of weight loss. The endothermic peak at ca. 97.4 °C is assigned to the loss of physically adsorbed water^30^. The peak at ca. 446.7 °C is ascribed to the weight loss of hydroxyl groups bonded with Al (-OH_Al-OH_), *i. e*. the dehydroxylation of AlOOH.

### Role of surface defects for hydrogenation

To further confirm the OH defects on the AlOOH surface, electron paramagnetic resonance (EPR) was used to study these catalysts. The EPR results shown in Fig. 4b suggest that there are two kinds of defects in the different chemical environments on the surface of AlOOH^34^, a OH vacancy (OH_v_) and a surface O site (O_Hv_) created by one H removed from OH. The fitting results of all samples are shown in Supplementary Fig. 4 and a hyperfine coupling from surrounding H species was observed in SysA with an A_H_-value of 53.69 MHz (Supplementary Table 4-1). Thus SysA, SysB and SysC could be assigned to Al-OH_v_-Al, Al-O_Hv_-Al and background signal, respectively. DFT calculation also shows that OH_v_ and O_Hv_ could form FLPs. A combination of EPR and XPS results revealed that the adsorbed water occupied the defects on the surface of AlOx-U1, AlOOH-U3 and AlOOH-U4, resulting in the decrease in activity. It is easier to construct FLPs with both defects on the catalyst surface.

**Fig. 5:**
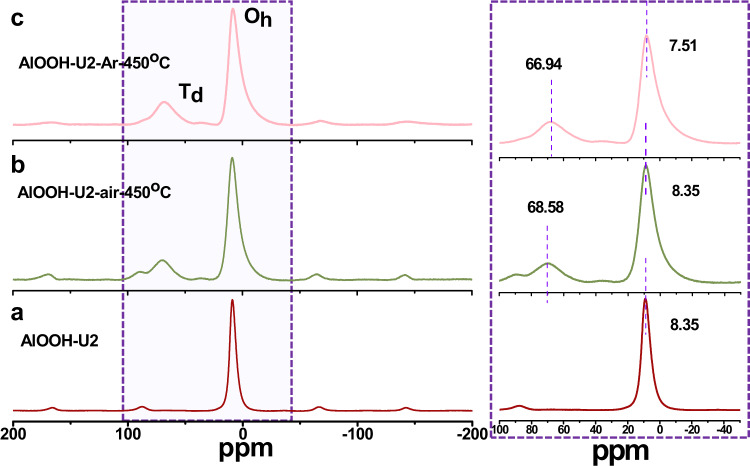
^27^Al solid NMR of AlOOH. Catalyst treated in different conditions. **a** AlOOH-U2. **b** AlOOH-U2-air-450 °C. **c** AlOOH-U2-Ar-450 °C.

To clarify the role of surface OH defects, some control experiments were conducted. Based on TGA analysis, the chemical bonds of Al-OH in AlOOH could be broken at ca. 450 °C. The catalyst AlOOH-U2 was calcined in air at 450 °C for 3 h, denoted as AlOOH-U2-air-450 °C. As the FTIR spectra shown in Fig. 4c (green line), two characteristic absorption peaks of AlOOH at ca. 3099 cm^−1^ and 1075 cm^−1^ associated with -OH_Al-OH_ disappear, implying that the chemical bonds of Al-OH in AlOOH was broken. The EPR of AlOOH-U2-air-450 °C (Supplementary Fig. 5) shows a *g* value of 2.003 with only one peak, indicating that the surface FLPs are locked by O after calcination in air. As shown in XRD patterns (Fig. 4d, green line), AlOOH-U2-air-450 °C exhibited an amorphous aluminum oxide structure. However, the layered structure of AlOOH-U2 was preserved for AlOOH-U2-air-450 °C as shown in Supplementary Fig. 2a. As expected, the catalytic activity of AlOOH-U2-air-450 °C was decreased significantly, (Table 1, entry 4). The decreased catalytic activity may be attributed to the disappearance of surface OH defects, which leads to the destruction of FLP sites. For comparison, a perfect FLP surface catalyst was prepared by treating AlOOH-U2 under Ar atmosphere at 450 °C for 3 h, denoted as AlOOH-U2-Ar-450 °C. The AlOOH-U2-Ar-450 °C also presents an amorphous alumina oxide structure (Fig. 4d, pink line). The FTIR spectra (Fig. 4c, pink line) also show that the stretching and bending vibration modes of -OH_Al-OH_ disappeared. However, a higher field chemical shift was observed in the ^27^Al NMR of AlOOH-U2-Ar-450 °C compared with AlOOH-U2-air-450 °C (Fig. 5). This was attributed to the electron shielding effects on the Al nucleus caused by abundant OH defects. As expected, the catalytic activity of AlOOH-U2-Ar-450 °C was higher than that of AlOOH-U2. As shown in Table 1. entry 6, complete conversion was achieved in 4 h. These experiments also provided evidence that the catalytic activity was related to the OH defects.

The Lewis acidic and basic sites in AlOOH-U2 were characterized by FT-IR of pyridine adsorption and CO_2_-TPD, and the results showed that there were both Lewis acidic and basic sites on the AlOOH-U2 surface (Supplementary Fig. 6). It has been reported that Lewis acidic sites could be destroyed by Lewis bases pyridine^21^. The catalytic activity of AlOOH-U2 was checked in the presence of pyridine and it was found that the activity of the catalyst was completely quenched (Table 1, entry 2). We also found that the catalyst was not active in the presence of benzoic acid since the Lewis basic sites were blocked by benzoic acid (Table 1, entry 3). These results demonstrated the important role of Lewis acidic and basic sites during the catalysis. Furthermore, no product was detected in H_2_O since H_2_O can destroy the structures of FLP sites by competitive adsorption on OH defect sites, resulting in serious deactivation of the catalyst. (Table 1, entry 1).

### Mechanism investigations

**Fig. 6:**
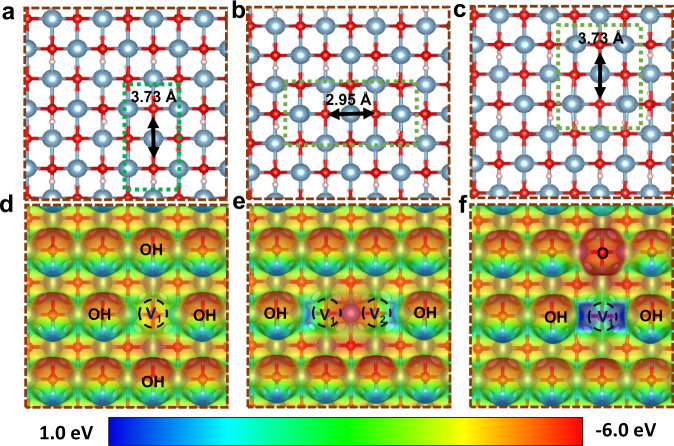
Images of possible constructed solid surface frustrated Lewis pairs on AlOOH (010). Optimized structure of AlOOH surface with one OH defect **a**, two OH defects **b** and one OH_v_ and O_Hv_ **c**. Electrondensity isosurface of AlOOH surface with one OH defect **d**, two OH defects **e** and one OH_v_ and O_Hv_ **f**. The color bar represents the electrostatic potential scale.

All above control experiments strongly suggest that the OH defects play extremely important roles in activating H_2_ and achieving high catalytic activity for hydrogenation reactions under mild conditions. To clarify how the OH defects construct FLP active sites on the AlOOH surface, the calculations were performed using spin polarized DFT methods as implemented in the QUICKSTEP code^35^ of the CP2K package^36^. Three slab models containing different OH defects were built as shown in Fig. 6. In the first model (Fig. 6a, d), a hydroxyl group in AlOOH (010) is removed, forming one OH vacancy (OH_v_) on the surface. The distance of OH_v1_ and OH is 3.73 Å in line with FLPs to activate molecular hydrogen, but only weak charge separation is observed. Two adjacent OH vacancies are constructed in model II (Fig. 6b, e), while the electrostatic potential in two vacancies is symmetric indicating no FLP site is formed in model II. Considering the non-reducibility of AlOOH, in model III (Fig. 6c, f) one hydroxyl and one hydrogen are removed on the (010) surface to construct one FLP site between the OH vacancy (OH_v_) and surface oxygen (O_Hv_). Compared to the model I, the absence of hydrogen increases the electron density of surface oxygen, meanwhile, the Lewis acidic site in OH vacancy is stronger than that of model I. Notably, the distance between OH_v1_ and O is 3.73 Å, the same as the distance between OH_v1_ and OH in model I.

**Fig. 7:**
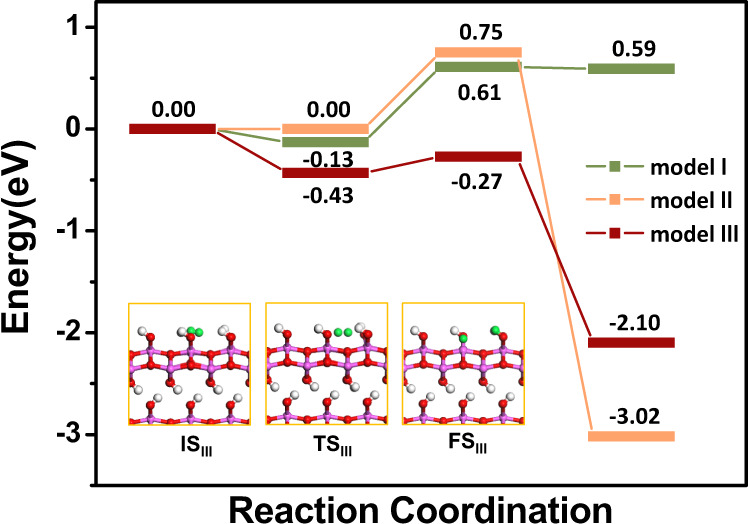
DFT calculations of activation of H_2_ molecules on AlOOH. The H_2_ adsorption and dissociation pathways on the surface of AlOOH in different models.

Hydrogen dissociation pathways on three models were also calculated using Cl-NEB method^37,38^. The energy profile is shown in Fig. 7. On the AlOOH (010) surface containing one OH defect (model I), the H_2_ was weakly adsorbed on OH defect sites with adsorption energies of -0.13 eV, and then dissociated over 0.74 eV energy barriers reaching the final states of 0.59 eV. Molecular H_2_ was not able to adsorb on the unsaturated Al sites in model II, and the energy barrier of dissociation was as high as 0.75 eV since no FLPs were formed in this model to activate H_2_. After H_2_ dissociation, two OH vacancies were filled with H species leading to the most stable final state among three models, of -3.02 eV. In model III, H_2_ was adsorbed on the FLP site with a strong adsorption energy of -0.43 eV, and the Hirshfeld population analysis^39^ (Supplementary Fig. 12) indicated that the H_2_ was evidently positive charge in the IS_III_. Moreover, the H-H bond in model III was longer than that of the other two models. The strong adsorption and stretched H_2_ bond demonstrated that molecular hydrogen was activated in FLP sites of model III. The energy barrier of H_2_ dissociation was as low as 0.16 eV, while the reaction was exothermic -2.10 eV.

Among the three hydrogen adsorption modes, the net charge of molecular hydrogen in model III was +0.613 *e*, while it was +0.267 *e* and +0.218 *e* in the other two modes (Supplementary Fig. 12). The net charge indicates that the Lewis acid site plays a vital role in AlOOH-based FLPs, which was also verified by Hirshfeld population analysis. As shown in Supplementary Table 5–Table 8, the charge on surface O species varied from -0.826 *e* to -1.012 *e*, however the charge on Al species near defeats was +0.204 *e* in model II and +1.126 *e* in model III (Supplementary Table 7, 8). Considering with small ion radius and high charge density of Al^3+^ species, its acidity increased sharply when Al coordination decreases. In model I, the removal of one OH group leads to a single electron localized in the defect (Supplementary Fig. 13), which decreased the Lewis acidity of exposed Al as shown in Fig. 6a.

In model III, removal of one H_2_O molecule in an appropriate distance from AlOOH (010) surface resulted in no localized single electron on the surface, while it increased the acidity of formed FLPs since Al’s orbitals in the defect was not occupied and the electron pair was localized in surface oxygen (O_Hv_). The twisted Al-O structures near the defect in model III also proved the unoccupied orbitals in the defect and enhanced Lewis acidity, compared to models I and II. The computational studies combined with characterizations have provided obvious evidence for the formation of FLPs on non-reducible oxides and their hydrogen activation ability.

According to our DFT calculations, H_2_ adsorbed on AlOOH was readily split into H^δ+^ and active H species with charges of 0.144 *e* and 0.015 *e*, respectively. One of the H^δ+^ boned to the Lewis base site O_Hv_ to yield O-H^δ+^, and the other active H species was stabilized by surrounding Al atoms. It indicates the mode of H_2_ splitting on AlOOH is heterolytic. To test the proposed mechanism, we explored the kinetic isotope effect (KIE) with D_2_ in styrene hydrogenation. Before the KIE experiment, we first confirmed that the reaction follows the first-order kinetics with respect to hydrogen pressure (Supplementary Fig. 14a). A primary isotope effect was observed (ratio of reaction rates using H_2_ and D_2_, k_H_/k_D_ = 2.97) implying the heterolytic dissociation of hydrogen^40^ (Supplementary Fig. 14b). In addition, the GC-MS analysis detected the ethylbenzene with a molecular weight (Mw) 107, indicating the addition of an H atom and a D atom to styrene (Supplementary Fig. 15). These results demonstrate that the H_2_ splitting over the AlOOH catalysts occurs via heterolytic mode.

We also notice that the surface hydroxyl groups are not rigidly restrained and may lead to a disordered catalytic surface. To valid the abovementioned mechanism, a disordered surface model was built with ‘heating and quenching’ techniques^41^. Since Model III FLP site exhibits the lowest hydrogen dissociation barrier, a similar FLP site is introduced in disorder models. (Supportmental Note 1). The calculated molecular hydrogen activation pathways on the disordered surface show similar reaction energies and barriers to the above ideal surface.

### Hydrogenation scope of alkenes and alkynes

The scope of hydrogenation reactions catalyzed by AlOOH-U2 was demonstrated in Table 2. The substrate conversion of 100% was achieved under 80 °C for the hydrogenation of *p*-methyl styrene and *p*-chlorostyrene (Table 2, entry 1 and entry 3). For *p*-methoxystyrene, a 92.8% conversion was obtained which was slightly lower than that of *p*-methyl styrene (Table 2, entry 2). The catalytic activity of AlOOH-U2 for 4-*tert*-butylstyrene and *trans*-stilbene was very low due to steric hindrance. The conversion of 4-*tert*-butylstyrene was only 37% at 80 °C (Table 2, entry 4). The conversion of *trans*-stilbene was still only 2.3% even by extending the reaction time to 24 h and increasing the temperature to 100 °C (Table 2, entry 6). The lower reactivity of 4-aminostyrene (Table 2, entry 1 and entry 5) could be attributed to the interaction between amino (-NH_2_) and Al^3+^_unsatur._. For the hydrogenation of phenylacetylene, styrene and ethylbenzene were yielded. A 100% conversion of phenylacetylene with 97.4% selectivity towards ethylbenzene was obtained under 3.0 MPa H_2_ pressure at 80 °C (Table 2, entry 7). A complete conversion but only 64.9% olefin selectivity was got for 4-methylphenylacetylene hydrogenation (Table 2, entry 8). For 1-chloro-4-ethynylbenzene and 4-ethinylaniline, the conversions were all over 90% and the selectivity of olefin was slightly higher than that of alkanes (Table 2, entry 9 and 10). For diphenylacetylene, 92.2% conversion was afforded and the selectivity of *cis*-stilbene and *trans*-stilbene was 92.4% and 7.6%, respectively (Table 2, entry 11).

**Table 2:** Scope of AlOOH-U2 for catalytic hydrogenation of alkenes and alkynes ^a^.

Entry	Substrate	Product	Temp./°C	Time /h	Pressure /MPa	Conv./%	Sel./%
1^*b*^			80	10	2	100	>99
2			80	10	2	92.8	>99
3			80	10	2	100	>99
4			80	10	2	37.0	>99
5			80	10	2	82.9	>99
6			100	24	2	2.3	>99
7			80	24	3	100	2.6 97.4
8			80	24	3	100	64.9 35.1
9^*b*^			80	24	3	92.5	80 20
10			80	24	3	92.0	53.8 46.1
11			80	24	3	92.2	92.4 7.6

^a^ **Reaction conditions**: substrate (0.2 mmol), THF (2 mL), AlOOH-U2 catalyst (50 mg), stirring speed (600 rpm); the substrate conversion and product selectivity were determined by GC with n-decane as internal standard

^b^ n-Dodecane as internal standard.

Furthermore, the reactivity of 4-vinylbenzaldehyde was also checked. Only 4-ethylbenzaldehyde was produced, with 54% yield and >99% selectivity. The lower reactivity of 4-vinylbenzaldehyde could be ascribed to the blockage of surface FLP sites on AlOOH by the strong adsorption of C = O at the OH vacancy. The DFT calculations further proved the result that the adsorption energies of C = O and C = C are -1.97 eV and -0.69 eV (Supplementary Fig. 16), respectively.

In summary, we constructed a steric independent O_Hv_ /Al^3+^_unsatur._ surface FLP sites on non-reducible layered AlOOH for H_2_ activation which could efficiently catalyze the hydrogenation of alkenes and alkynes with a wide scope under mild conditions (80 °C, 2 MPa). Based on DFT calculations, the H_2_ dissociation barrier is as low as 0.16 eV. It is demonstrated that the rich OH defects over AlOOH are the key to generating Lewis base and Lewis acid center. In addition, the layered structure of AlOOH exposes more defects, resulting in a high concentration of O_Hv_ /Al^3+^_unsatur._ FLPs, which are conducive to highly efficient hydrogen activation.

## Methods

### Catalyst preparation

Layered AlOOH were prepared by the solvothermal method. In a typical experiment, 20 mmol Al (NO_3_)_3_·9H_2_O and a certain amount of urea was dissolved in 30 mL deionized water and 30 mL ethylene glycol, respectively. And then the Al (NO_3_)_3_ solution was added into the urea solution. After vigorously stirring at room temperature for an hour, the mixture was transferred into a 100 mL Teflon-lined stainless-steel autoclave, followed by a solvothermal treatment at 180°C for 5 h. After being cooled to room temperature, the white translucent gel was separated by centrifugation, washed with deionized water several times until the pH was about 7, and then dried in an oven at 80°C overnight. The final white catalysts were obtained by calcination under an air atmosphere at 350 °C for 4 h. The obtained catalysts were denoted as AlOOH-UX, where X refers to the ratio of urea and Al precursor. The catalysts are ground with agate mortar before use.

### Catalyst Characterization

The powder X-ray diffraction spectra (Power XRD) measurements were performed on a Rigaku D/max 2500 instrument using Cu Kα radiation (50 kV). Transmission electron microscopy (TEM) images were obtained on an HT7700 electron microscope at an acceleration voltage of 120 kV. The element energy dispersive spectroscopy (EDS) mappings were operated at 200 kV on a JEOL-2100F electron microscope. The high-resolution TEM images were obtained at 200 kV on JEM-F200. X-ray photoelectron spectroscopy (XPS) was performed on the Thermo Scientific ESCALab 250Xi using 200 W monochromatic Al Kα radiation. The hydrocarbon C1s line at 284.8 eV from adventitious carbon is used for energy referencing. FT-IR spectra of the catalysts with pyridine absorbed on the catalysts were recorded with a Bruker TENSOR 27 spectrometer. The sample (50 mg) of AlOOH-U2 catalyst was dispersed in a THF solution of pyridine at 80 °C and stirred for 8 h. The suspension was centrifuged and washed with THF to remove the physically absorbed species, and then dried at 60 °C for 12 h. The catalysts were blended with KBr for IR characterization. Thermogravimetric analysis (TGA) was done using a Netzsch Germany STA449F3 instrument in N_2_ atmosphere at a heating rate of 10 °C min^−1^ from room temperature to 1000 °C. The products were analyzed quantitatively by a gas chromatograph (Agilent 7890B) equipped with a flame ionization detector (FID). Identification of the products and reactant was done using a GC–MS (Shimadzu QP2010, DB-5MS capillary column) as well as by comparing the retention time with n-decane as the internal standard used in GC traces. The EPR data were gained from Bruker EMXplus-9.5/12. ^27^Al NMR spectra were recorded on a JNM-ECZ600R 600 spectrometer equipped with a 3.2 mm tube diameter. The corresponding resonance frequency and magic angle spinning rate were 156 MHz and 12 kHz respectively. ^27^Al NMR measurements were performed without any pretreatment on the samples.

### Catalytic activity tests

The reaction was carried out in a Teflon-lined stainless-steel reactor of 16 mL with a magnetic stirrer. In a typical experiment, the desired amount of catalyst, reactant and solvent were added into the reactor. The air in the reactor was replaced with H_2_ for 3 times and then the reactor was pressurized with H_2_ to a certain pressure. The reactor was put into an air bath at the desired temperature. After a certain reaction time, the autoclave was placed into an ice-water mixture, cooled to room temperature, and a known amount of internal standard n-decane was then added to the reactor. Then the reaction solution was centrifuged to separate the catalyst and the liquid was analyzed by GC analysis. The conversion of reactants and the selectivity of the products were calculated based on the GC data. The reaction experiments of other substrates are the same as those mentioned above.

### Computational details

All DFT calculations were performed using QUICKSTEP code^35^ of CP2K package^36^. The generalized gradient approximation (GGA) with Perdew-Burke-Ernzerhof (PBE) functional was used to evaluate the exchange and correlation^42^. The wave functions were expanded in a molecularly optimized (MOLOPT) double-ζ Gaussian basis set^43^ with a cutoff energy of 450 Ry and Goedecker–Teter–Hutter pseudopotentials^44^. The empirical dispersion correction (Grimme D3) within a range of 15 Å was used to correct dispersion effects^45^. All transition states were located with the climbing image NEB method with a force convergence limit of 0.03 eV/Å.

The two-layer p (5 × 4) slab models were based on boehmite crystal structure, and the most stable (010) facet was selected to simulate the catalyst surface. The bottom layer was fixed during geometry optimization to mimic the bulk phase. A 15 Å thick vacuum layer was induced to eliminate the interaction between neighboring slabs.

## Supplementary information

Supplementary Information

## Data Availability

The primary data that support the plots within this paper and other finding of this study are available from the corresponding author on reasonable request.
