# Supplementary material for: Solid surface frustrated Lewis pair constructed on layered AlOOH for hydrogenation reaction
Source: Nat Commun. 2022 Apr 28;13:2320. doi: 10.1038/s41467-022-29970-6 (PMC9050862; doi:10.1038/s41467-022-29970-6)
Supplement: Supplementary file 1 — Supplementary Information [file 41467_2022_29970_MOESM1_ESM.pdf]

Supplementary Information

Solid Surface Frustrated Lewis Pair Constructed on Layered AlOOH for Hydrogenation Reaction

Shulin Liu,[1,2,3] Minghua Dong,[1,2,3] Yuxuan Wu,[1,2] Sen Luan,[1,2] Yu Xin,[1,2] Juan Du,[1,2] Shaopeng Li, [1,2] Huizhen Liu,[1,2]* Buxing Han[1,2]

1. Beijing National Laboratory for Molecular Sciences, CAS Key Laboratory of Colloid and Interface and Thermodynamics, Institute of Chemistry, Chinese Academy of Sciences, Beijing 100190, China.
2. School of Chemistry and Chemical Engineering, University of Chinese Academy of Sciences, Beijing 100049, China.
3. Both authors contributed equally.

Supplementary Methods
Supplementary Figure 1 HR-TEM images of AlOOH-U3 , AlOOH-U4, and the inserting as FFT images.
Supplementary Figure 2 TEM images and the XRD patterns of AlOOH-U2.
Supplementary Figure 3 FTIR analysis of AlOOH-U2 after H_2 treatment.
Supplementary Figure 4 EPR fitting of all catalysts. The color of lines: black- experimental data, purple-fitting line.
Supplementary Figure 5 EPR analysis of AlOOH-U2 after air treatment.
Supplementary Figure 6 Characterization of acid-base properties on AlOOH-U2.
Supplementary Figure 7 EPR analysis of AlOOH-U2 after Ar treatment.
Supplementary Figure 8 AlOOH Computional model.
Supplementary Figure 9 Side views structures of H_2 dissociation in model I, II and III.
Supplementary Figure 10 Structure Models of different AlOOH surface.
Supplementary Figure 11 DFT calculations of activation of H_2 molecules on AlOOH.
Supplementary Figure 12 Hirshfeld population charge analysis.
Supplementary Figure 13 Electron spin density.
Supplementary Figure 14 Results of deuterium kinetics experiments.
Supplementary Figure 15 Mass spectra of product in different gas.
Supplementary Figure 16 Adsorption energy calculation of p-formylstyrene.
Supplementary Table 1 Energies of the differently oriented surfaces.
Supplementary Table 2 Optimization of styrene hydrogenation catalyzed by AlOOH
Supplementary Table 3 Results of XPS analysis of O1s of the AlOOH catalysts
Supplementary Table 4 Fitting parameters of EPR
Supplementary Table 5 Hirshfeld-I and Mulliken Population Analysis of intact AlOOH (010)
Supplementary Table 6 Hirshfeld-I and Mulliken Population Analysis of OH_v AlOOH (010)
Supplementary Table 7 Hirshfeld-I and Mulliken Population Analysis of $2OH_v$ AlOOH (010)
Supplementary Table 8 Hirshfeld-I and Mulliken Population Analysis of OH_v and O_{Hv} AlOOH (010)
Supplementary Table 9 Compare cell parameters in computational simulations, experiments and literature
Supplementary Table 10 ssFLP distance in different models and corresponding activation barriers

Supplementary Methods

Chemicals. Ethylene glycol (99%), urea (99.3+%, Alfa Aesar), styrene (99.5%, stab. with HQ)，4-Methylstyrene (98+%, stab. with 0.1% 3,5-di-tert-butylcatechol, Alfa Aesar), 4-Ethyltoluene (97%, Alfa Aesar), 1-Methoxy-4-vinylbenzene (98%), 4-Ethylanisole (97%+, Adamas), 4-tert-butylstyrene (94%, stab. with 50 ppm 4-tert-butylcatechol), 4-Aminostyrene (97%, Alfa Aesar), 4-Ethylaniline (98+%), Phenylacetylene (97%), Diphenylacetylene (99%), 4-Chlorostyrene (97%, with TBC), 1-Chloro-4-ethylbenzene (97%, Aladdin), 1,2-diphenylethane (98%+, Adamas), trans-Stilbene (98%, Aladdin), cis-Stilbene (97%), 1-Chloro-4-ethynylbenzene (98%), 4-Ethynyltoluene (98%, Aladdin) 4-Ethinylaniline (97%, Acros) n-Butanol (99.5%), 1-Pentanol (99%) were purchased from Innochem Chemical Reagent Company. $Al(NO_3)_3 \cdot 9H_2O$ (99.5%) were purchased from Sinopharm Chemical Reagent Co., Ltd. D_2O (99.8%), n-decane (98.5%) and n-dodecane (98.5%) were purchased from J&K Company. Ethanol (99.8%, AR grade) were bought from Beijing Chemical Reagent Factory. All the chemicals were used as received without any further purification.

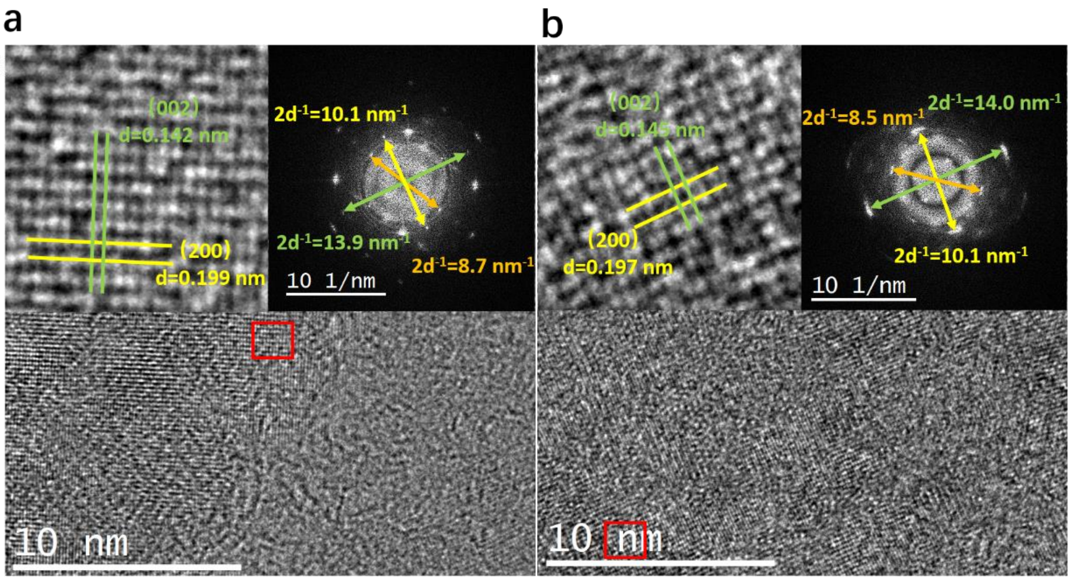

Supplementary Figure 1. HR-TEM images of AlOOH-U3 , AlOOH-U4, and the inserting as FFT images. (a) AlOOH-U3. (b) AlOOH-U4.

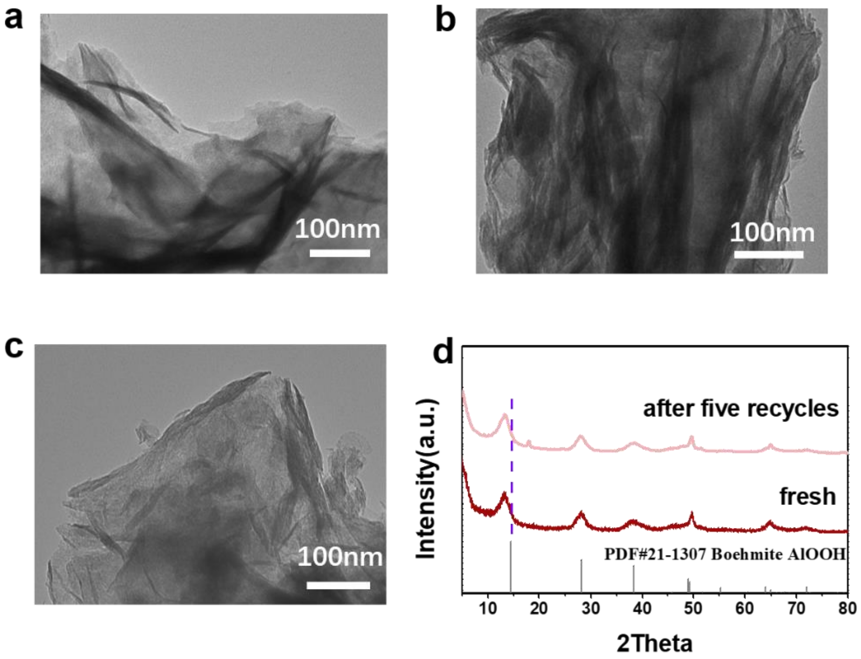

Supplementary Figure 2. TEM images and the XRD patterns of AlOOH-U2. (a) AlOOH-U2-air-450°C. (b) AlOOH-U2 after five times recycles. (c) AlOOH-U2-Ar-450°C. (d)XRD of AlOOH-U2 after five recycles.

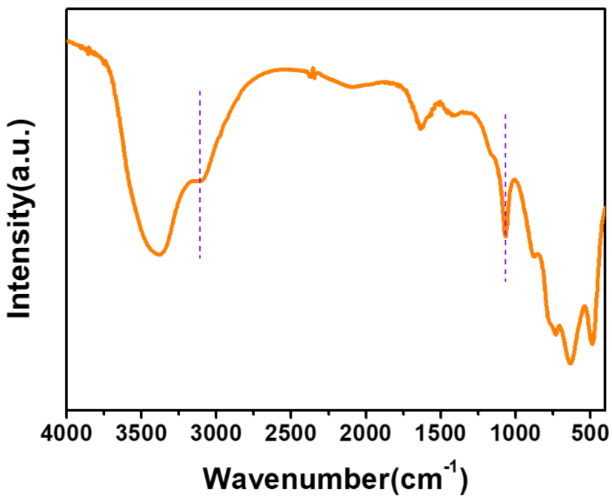

Supplementary Figure 3. FTIR analysis of AlOOH-U2 after H_2 treatment. FTIR spectra of AlOOH-U2-H_2-300ºC.

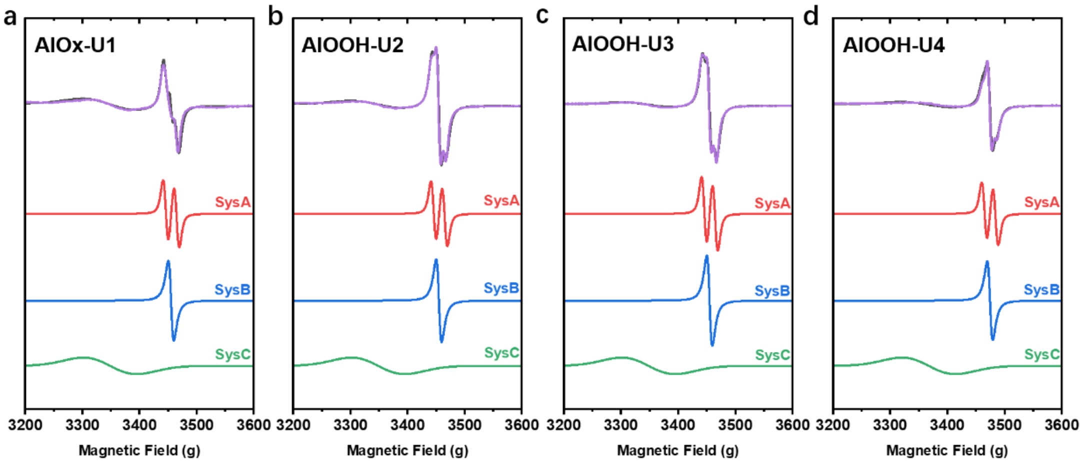

Supplementary Figure 4. EPR fitting of all catalysts. (a) AlOx-U1. (b) AlOOH-U2. (c) AlOOH-U3. (d) AlOOH-U4. The color of lines: black- experimental data, purple-fitting line.

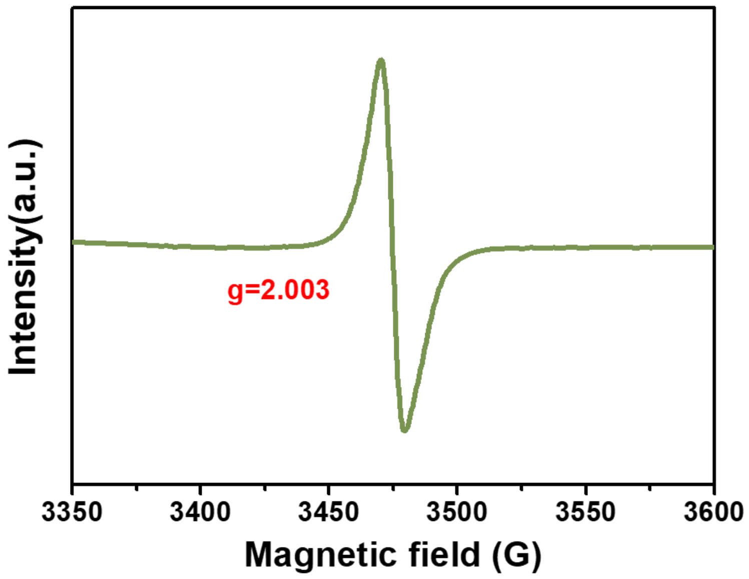

Supplementary Figure 5. EPR analysis of AlOOH-U2 after air treatment. EPR of AlOOH-U2-air-450°C.

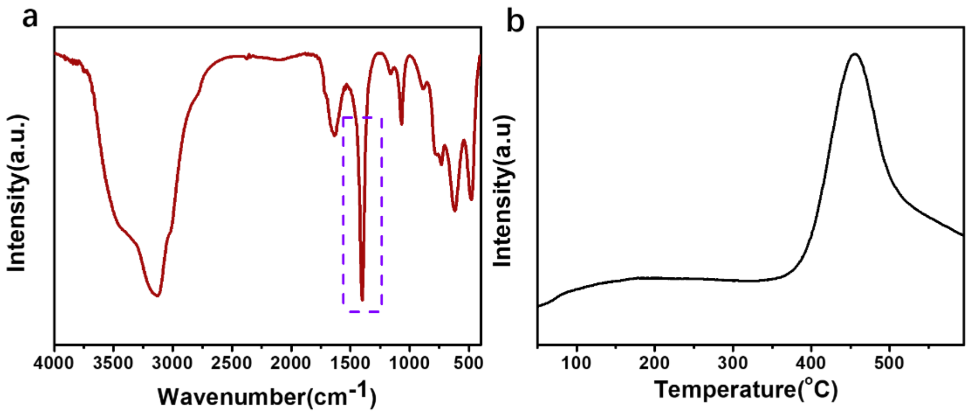

Supplementary Figure 6. Characterization of acid-base properties on AlOOH-U2. (a) FT-IR spectra of pyridine adsorption on AlOOH-U2. (b) CO_2-TPD of AlOOH-U2 with a cooling well.

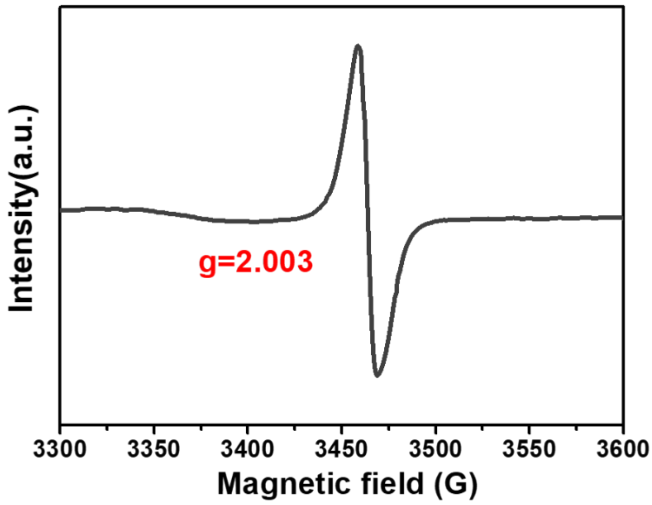

Supplementary Figure 7. EPR analysis of AlOOH-U2 after Ar treatment. EPR of AlOOH-U2-Ar-450°C.

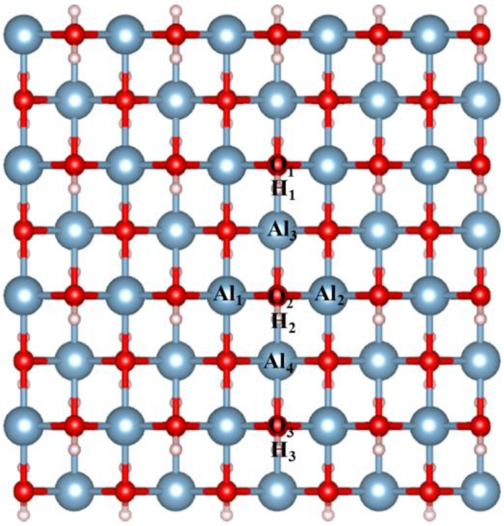

Supplementary Figure 8. AlOOH Computional model. Intact p(5×4) (010) surfaces.

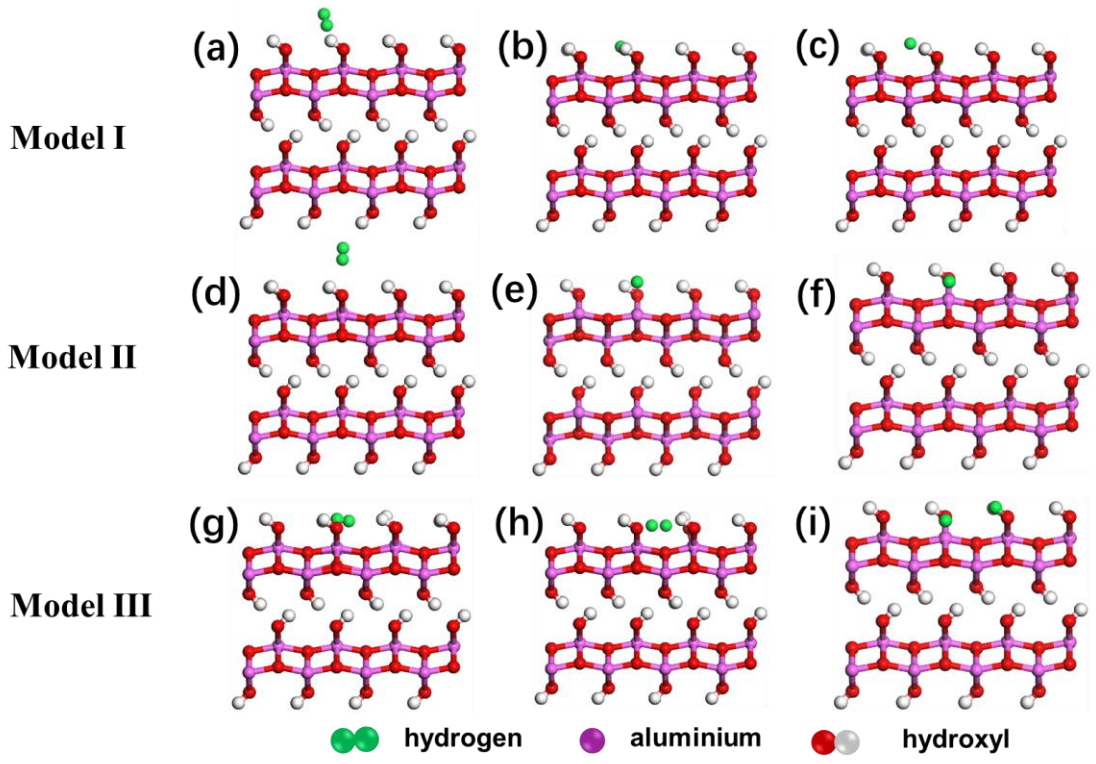

Supplementary Figure 9. Side views structures of H_2 dissociation in model I, II and III. (a) IS (b) TS and (c) FS in model I. (d) IS (e) TS and (f) FS in model II. (g) IS (h) TS and (i) FS in model III.

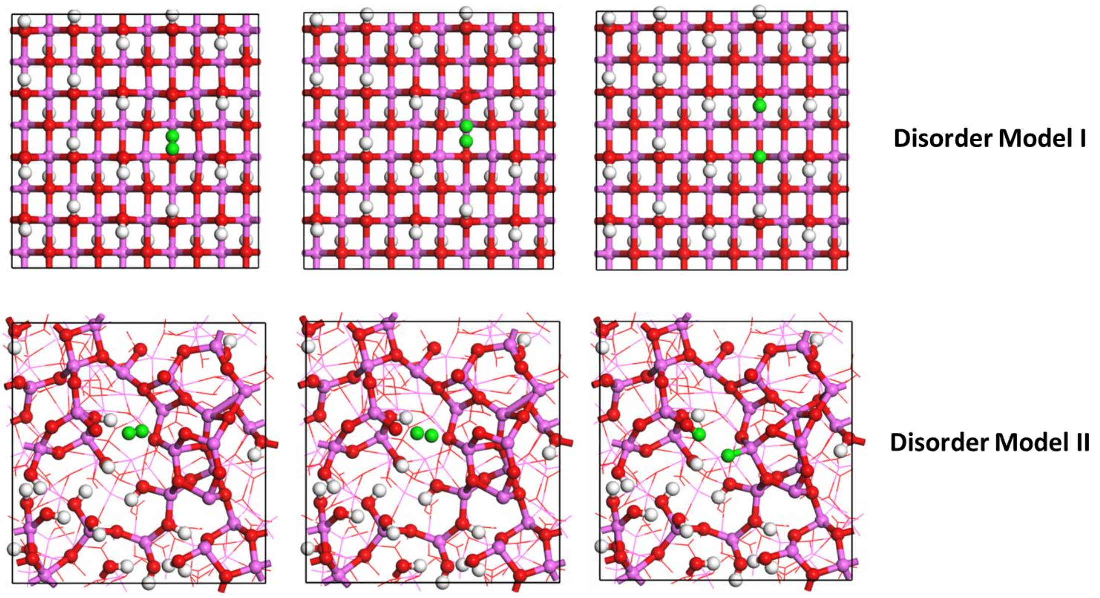

Supplementary Figure 10. Structure Models of different AlOOH surface. Key structures in H_2 dissociation pathways on the disordered surface of AlOOH.

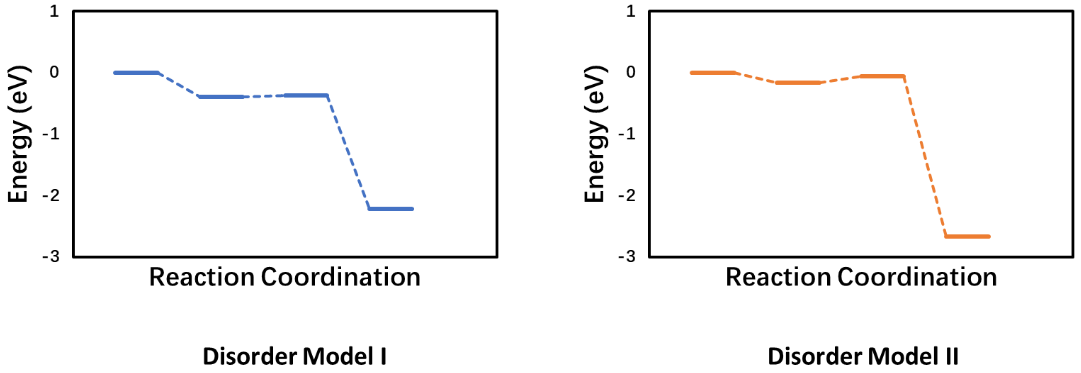

Supplementary Figure 11. DFT calculations of activation of H_2 molecules on AlOOH. The H_2 adsorption and dissociation pathways on the disordered surface of AlOOH.

Supplementary Note 1

Herein, we consider the disordered model from both experimental and computational aspects. The disordered model I is built by the 'heating and quenching' technique. The two-layer p (5×4) slab models with 15 Å vacuum layer is the initial configuration. 800K, a little higher than experimental calcination temperature, was chosen to perform AIMD simulations. However, no obvious reconstruction of Al-O framework is observed in our MD trajectory, probably because the (010) surface is the most stable AlOOH surface. After thermal equilibration, the whole system is quenched to 0 K by velocity rescaling method in 1.5 ps at a quenching rate ≈ 5×10^{14} K/s, and then optimized to obtain the ground state.

To justify our model, structure parameters are calculated from the optimized structure and compared with experimental values (Supplementary Table 9). We note that all MD simulations are performed in NVT ensemble, thus the cell parameters, *a* and *c*, are fixed as the chosen boehmite crystal

structure. The distance between two Al-O-H layers is relaxed, which is corresponded to cell parameters *b*. Thus, *b* is calculated as two times of distance between two Al-O-H layers The cell parameters also can be calculated from XRD data and HRTEM images. From the cell parameters, the density of boehmite is calculated as 3.10 g/cm^3, consistent with the literature[3].

Based on this disordered boehmite (010) surface produced by the 'heating and quenching' technique, we examine our previous mechanistic results. A similar Model III defect is introduced, and the molecular hydrogen activation pathway is calculated. The results show that the reaction energy is -2.23 eV, while the reaction barrier is calculated as low as 0.02 eV.

Furthermore, a computational disordered model II built by the 'melting and quenching' technique results in bulk disorder. The initial configuration is 5 × 1 × 4 AlOOH supercell, with cell parameters a = 3.71 Å, b = 13.12 Å and c = 2.89 Å and density of 2.83 g/cm^3. The large b is set to be consistent with our XRD results, and a relatively small density is obtained. The classical force field molecular dynamics is performed by LAMMPS software[2] with ClayFF[1]. The system is heating to 2500 K, higher than Al_2O_3 melting points. After reaching 2500 K, the system is quenched to 300 K at a quenching rate ≈ 2 ×10^{13} K/s and then heated to 2500 K again. Five 'melting and quenching' cycles are done, followed by geometry optimization at the DFT level to obtain the ground state. The dangling bonds on the surface are removed carefully in the final slab model, as shown in Supplementary Fig. 10. It can be seen that tetrahedral Al dominates in this disorder model, while in ideal boehmite crystal only octahedral Al exists. One model III defect is introduced, the hydrogen activation pathway is calculated. The reaction energy is -2.67 eV and the activation barrier is 0.08 eV. Thus we can conclude that the solid surface Lewis acid-base pair on AlOOH consists of surface oxygen and an unsaturated Al site and our computational results indicated that the arbitrary orientation of surface hydroxyl and bulk disordered Al-O framework has no significant influence in the ssFLP hydrogenation mechanism.

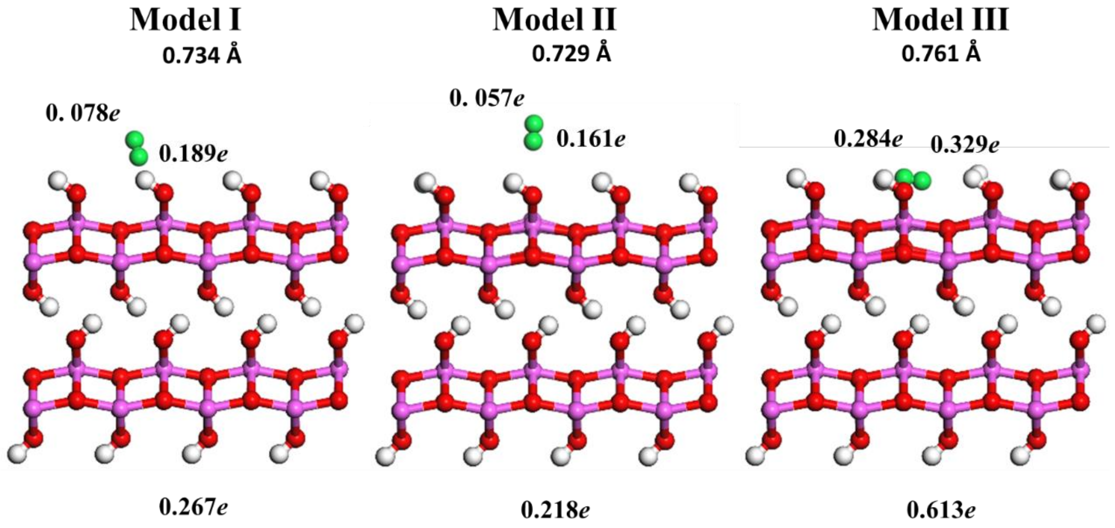

Supplementary Figure 12. Hirshfeld population charge analysis. H-H bond lengths and Hirshfeld population charge analysis of three H_2 adsorption configurations.

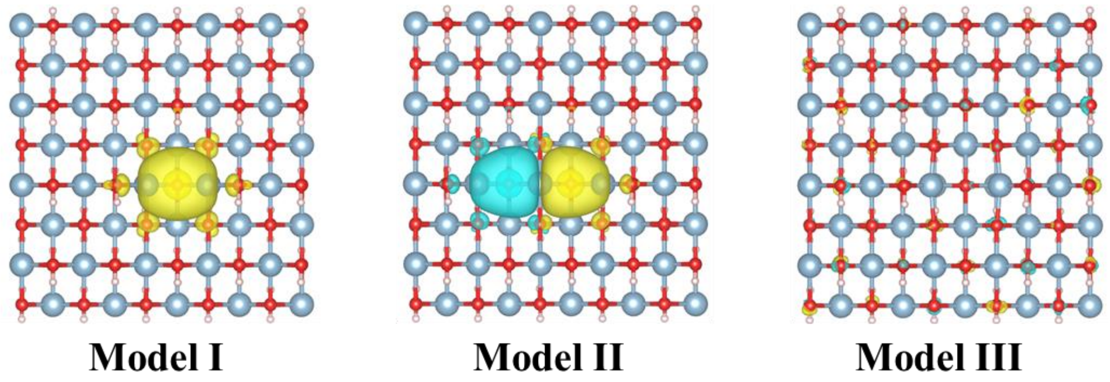

Supplementary Figure 13. Electron spin density. Spin density isosurfaces of model I, II and III.

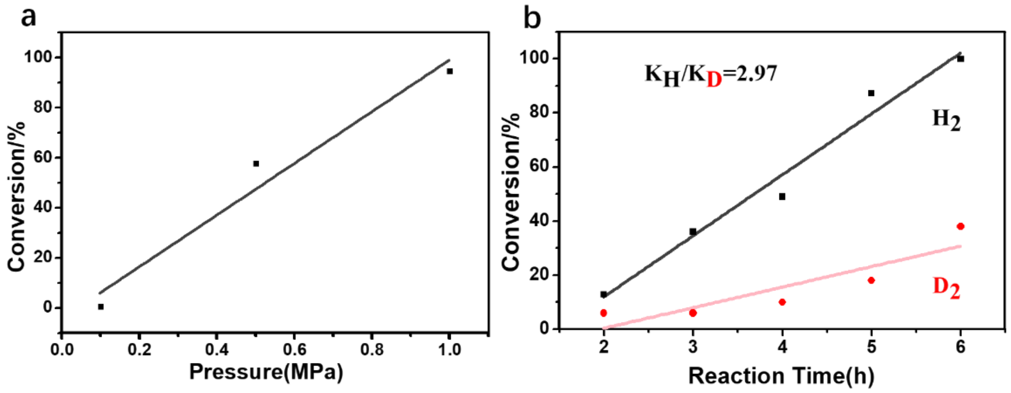

Supplementary Figure 14. Results of deuterium kinetics experiments. (a) Pressure curve of styrene hydrogenation. (b) Primary isotope effect observed for AlOOH-U2 in styrene hydrogenation.

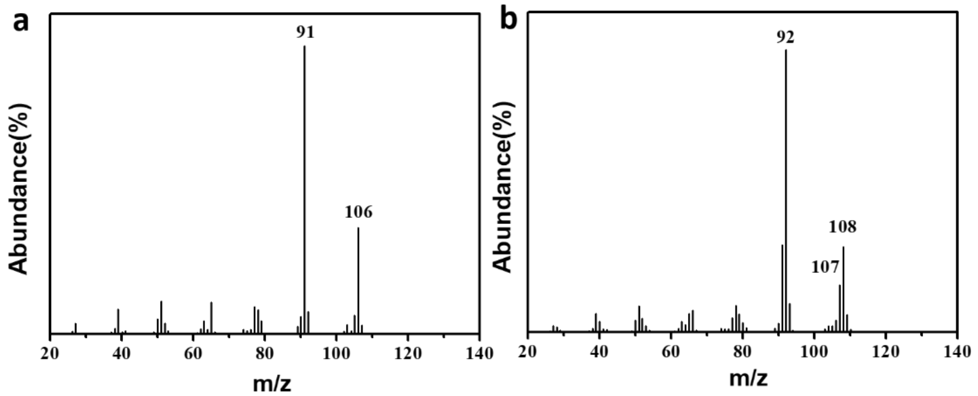

Supplementary Figure 15. Mass spectra of product in different gas. (a) Reaction in H_2. (b) Reaction in D_2.

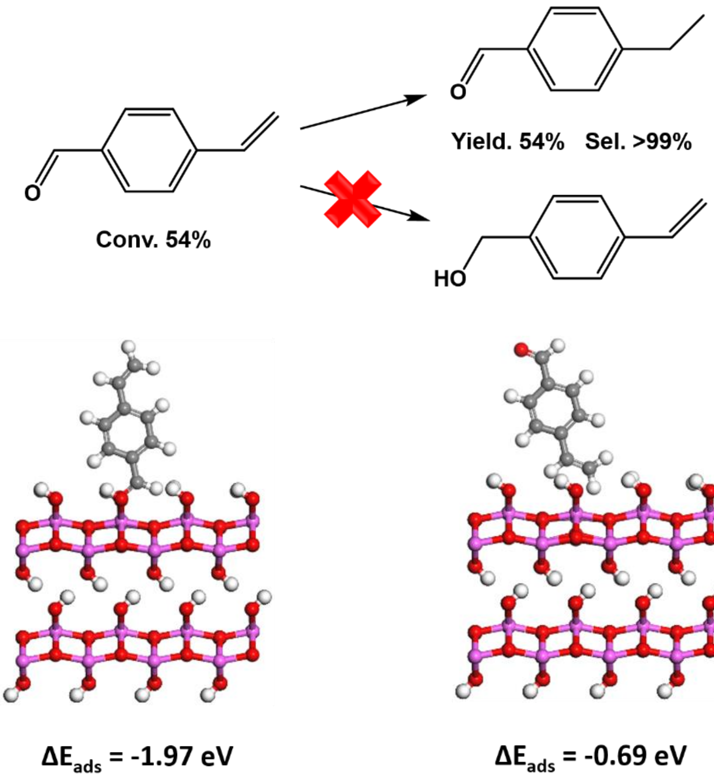

Supplementary Figure 16. Adsorption energy calculation of p-formylstyrene. Two adsorption configurations of p-formylstyrene on AlOOH (model III) and corresponding adsorption energies.

Supplementary Table 1. Energies of the differently oriented surfaces.

Facet	Surface energy [mJ/m^2]
(010)	398.2319
(100)	1428.138
(001)	2266.099

Supplementary Table 2. Optimization of styrene hydrogenation catalyzed by AlOOH

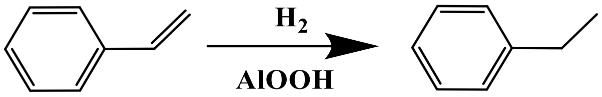

Entry	Catalyst	Temperature /°C	Time /h	Pressure /MPa	Conv. /%	Sel. /%
1	AlOx-U1	80	10	2	21.9	>99
2	AlOOH-U2	80	10	2	100.0	>99
3	AlOOH-U3	80	10	2	99.0	>99
4	AlOOH-U4	80	10	2	48.8	>99
5	AlOOH-U2	80	2	2	12.9	>99
6	AlOOH-U2	80	4	2	49.0	>99
7	AlOOH-U2	80	6	2	100.0	>99
8	AlOOH-U3	80	4	2	12.0	>99
9	AlOOH-U2	80	8	2	100.0	>99
10	AlOOH-U2	60	6	2	40.8	>99
11	AlOOH-U2	80	6	0.1	0.4	>99
12	AlOOH-U2	80	6	0.5	57.5	>99
13	AlOOH-U2	80	6	1.0	94.5	>99

Reaction conditions: styrene (0.5 mmol), THF (2 mL), AlOOH catalyst (50 mg), stirring speed (600 rpm) and n-decane as internal standard.

Supplementary Table 3. Results of XPS analysis of O1s of the AlOOH catalysts

Entry	Catalyst	O_{adH2O}/ O_{Total} (%)
1	AlOx-U1	39.4
2	AlOOH-U2	14.5
3	AlOOH-U3	21.6
4	AlOOH-U4	32.9

Supplementary Table 4-1. Fitting parameters of SysA

Catalyst	g	lwpp1	lwpp2	A	weight
AlOx-U1	2.0030	0.635	0.544	53.69	0.2603
AlOOH-U2	2.0030	0.635	0.544	53.69	0.2801
AlOOH-U3	2.0030	0.635	0.544	53.69	0.4772
AlOOH-U4	2.0030	0.635	0.544	53.69	0.1620

Supplementary Table 4-2. Fitting parameters of SysB

Catalyst	g	lwpp1	lwpp2	weight
AlOx-U1	2.0032	0	0.9581	0.2764
AlOOH-U2	2.0032	0	0.9581	0.8188
AlOOH-U3	2.0032	0	0.9581	1.039
AlOOH-U4	2.0032	0	0.9581	0.7781

Supplementary Table 4-3. Fitting parameters of SysC

Catalyst	g	lwpp1	weight
AlOx-U1	2.067	9.3367	1
AlOOH-U2	2.067	9.3367	1
AlOOH-U3	2.067	9.3367	1
AlOOH-U4	2.067	9.3367	1

Supplementary Table 4-4. The weight of SysA and SysB

Catalyst	SysA	SysB
AlOx-U1	48.5%	51.5%
AlOOH-U2	25.5%	74.5%
AlOOH-U3	31.5%	68.5%
AlOOH-U4	17.2%	82.8%

Supplementary Table 5. Hirshfeld-I and Mulliken Population Analysis of intact AlOOH (010)

Atom	Hirshfeld Charge (*e*)	Mulliken Charge (*e*)
Al_1	+0.759	+1.155
Al_2	+0.760	+1.155
Al_3	+0.760	+1.155
Al_4	+0.760	+1.155
O_1	-1.010	-0.543
O_2	-1.010	-0.543
O_3	-1.010	-0.543
H_1	+0.710	+0.147
H_2	+0.710	+0.147
H_3	+0.710	+0.147

Supplementary Table 6. Hirshfeld-I and Mulliken Population Analysis of OH_v AlOOH (010)

Atom	Hirshfeld Charge (*e*)	Mulliken Charge (*e*)
Al_1	+0.760	+0.939
Al_2	+0.759	+0.938
Al_3	+1.086	+1.144
Al_4	+1.080	+1.137
O_1	-1.012	-0.542
O_2		
O_3	-0.997	-0.536
H_1	+0.708	+0.144
H_2		
H_3	+0.709	+0.145

Supplementary Table 7. Hirshfeld-I and Mulliken Population Analysis of 2OH$_v$ AlOOH (010)

Atom	Hirshfeld Charge (*e*)	Mulliken Charge (*e*)
Al_1	+0.204	+0.692
Al_2	+0.966	+0.950
Al_3	+1.086	+1.124
Al_4	+1.083	+1.108
O_1	-1.011	-0.542
O_2		
O_3	-0.997	-0.534
H_1	+0.709	+0.144
H_2		
H_3	+0.709	+0.146

Supplementary Table 8. Hirshfeld-I and Mulliken Population Analysis of OH_v and O_{Hv} AlOOH (010)

Atom	Hirshfeld Charge (*e*)	Mulliken Charge (*e*)
Al_1	+1.123	+1.068
Al_2	+1.126	+1.067
Al_3	+1.079	+1.122
Al_4	+1.081	+1.136
O_1	-0.826	-0.676
O_2		
O_3	-1.003	-0.541
H_1		
H_2		
H_3	+0.711	+0.153

Supplementary Table 9. Compare cell parameters in computational simulations, experiments and literature

Entry	*a* (Å)	*b* (Å)	*c* (Å)	Reference
1	3.71	11.96	2.89	Disorder model I
2	3.71	13.12	2.89	Disorder model II
3	3.68	13.12	2.88	XRD results
4	3.94	-	2.88	HRTEM results
5	3.71	11.70	2.86	4

Supplementary Table 10. ssFLP distance in different models and corresponding activation barriers

Computation Model	ssFLP Distance (Å)	Activation Barrier (eV)
**Model I**	**3.73**	**0.74**
**Model II**	**2.95**	**0.75**
**Model III**	**3.73**	**0.16**
**Disorder Model I**	**3.63**	**0.02**
**Disorder Model II**	**4.56**	**0.08**

Supplementary Reference

1. Cygan, R. T., Liang, J. J. & Kalinichev, A. G. Molecular models of hydroxide, oxyhydroxide, and clay phases and the development of a general force field. *J. Phys. Chem. B* **108**, 1255-1266 (2004).
2. Plimpton, S. Fast Parallel Algorithms for Short-Range Molecular Dynamics. *J. Comput. Phys.* **117**, 1-19 (1995).
3. Wilson, S. J.,Stacey, M. H. The porosity of aluminum oxide phases derived from well-crystallized boehmite: Correlated electron microscope, adsorption, and porosimetry studies. *J. Colloid Interface Sci.* **82**, 507-517 (1981).
4. Motta, A., *et al*. Ab initio molecular dynamics study of the AlOOH boehmite/water interface: Role of steps in interfacial grotthus proton transfers. *J. Phys. Chem. C* **116**, 12514-12524 (2012).
